# Evacuation of Vertebral and Psoas Abscesses

**Published:** 1890-03-08

**Authors:** 


### EVACUATION OF VERTEBRAL AND PSOAS ABSCESSES.

Dr. Dollinger, of Buda-Pesth, in a rejoinder to Professor Lorenz's condemnation of operative treatment, says: "When I find a patient with inflammatory disease of the vertebræ of the lower dorsal and lumbar regions I look for psoas abscess." In children, at any rate, the smallest abscess can be detected by palpation: the hand laid on the abdomen over the iliac fossa, with the fingers pointed towards the umbilicus, the whole anterior aspect of the lumbar vertebral column, the psoas muscles on each side, and the inner surface of the iliac bones may be thoroughly explored. Sometimes, however, the abscess is in the substance of the psoas muscle, sometimes it reaches to the middle of the linea arcuata, or fills the iliac fossa without rising above the margin of the bone; or, again, it may ascend to the level of the umbilicus without protruding below Poupart's ligament. "If there are along with an abscess any signs of inflammation in the vertebra I open the abscess forthwith." He then describes his mode of procedure. Opening the abscess anteriorly, he wipes off the pyogenic membrane from the cavity of the abscess with the finger enveloped in sublimated cotton-wool, and then makes a counter opening posteriorly behind the outer margin of the quadratus lumbarum, and introduces a drainage tube. He then closes the anterior wound with sutures up to the lumen of the tube, and after washing out the cavity with sublimate solution, applies an antiseptic bandage. If this be well adjusted the subsequent discharge is rarely profuse. The only objection that can be urged against this procedure is the possibility of establishing a fistula, but this is rather an advantage, for the drying of the tuberculous focus really aids its cure. Should the inflammatory process in the vertebra have subsided he contents himself with opening and completely evacuating the abscess from before.

